# Formation Tracking Control and Obstacle Avoidance of Unicycle-Type Robots Guaranteeing Continuous Velocities

**DOI:** 10.3390/s21134374

**Published:** 2021-06-26

**Authors:** Jose Bernardo Martinez, Hector M. Becerra, David Gomez-Gutierrez

**Affiliations:** 1 Centro de Investigación en Matemáticas (CIMAT), Jalisco S/N, Guanajuato, Gto. 36023, Mexico; jose.morales@cimat.mx; 2 Multi-agent Autonomous Systems Lab, Intel Labs, Intel Tecnología de México, Av. del Bosque 1001, Zapopan, Jalisco 45019, Mexico; david.gomez.g@ieee.org; 3 Tecnologico de Monterrey, Escuela de Ingeniería y Ciencias, Av. General Ramón Corona 2514, Zapopan, Jalisco 45201, Mexico

**Keywords:** formation control, consensus, multi-agent system, hierarchical task-based control, unicycle-type robots

## Abstract

In this paper, we addressed the problem of controlling the position of a group of unicycle-type robots to follow in formation a time-varying reference avoiding obstacles when needed. We propose a kinematic control scheme that, unlike existing methods, is able to simultaneously solve the both tasks involved in the problem, effectively combining control laws devoted to achieve formation tracking and obstacle avoidance. The main contributions of the paper are twofold: first, the advantages of the proposed approach are not all integrated in existing schemes, ours is fully distributed since the formulation is based on consensus including the leader as part of the formation, scalable for a large number of robots, generic to define a desired formation, and it does not require a global coordinate system or a map of the environment. Second, to the authors’ knowledge, it is the first time that a distributed formation tracking control is combined with obstacle avoidance to solve both tasks simultaneously using a hierarchical scheme, thus guaranteeing continuous robots velocities in spite of activation/deactivation of the obstacle avoidance task, and stability is proven even in the transition of tasks. The effectiveness of the approach is shown through simulations and experiments with real robots.

## 1. Introduction

Wheeled mobile robots have been one of the most studied robotic platforms due to their applicability and motion capabilities. In the field of multi-agent systems (MAS), control schemes for collaborative wheeled robots have been developed for environmental sensing [1], task allocation for search and rescue [2], coverage control for precision agriculture [3] and object transportation [4], among others. For such applications, different control protocols have been reported in the literature, using centralized methods that depend on a global coordinate system as a first attempt, e.g., [5,6]. In these works, a virtual structure approach is used, where the complete formation is treated as a single rigid body and the motion of the formation is prespecified, i.e., given the trajectory of the formation centroid in global coordinates, the desired motion of each robot is obtained and an individual feedback controller corrects deviations.

Decentralized or distributed control methods are more robust to failures since only local interactions between agents of a network are required [7,8]. Consensus theory has become an essential tool to develop distributed control protocols [9]; the control laws based on consensus rely on the relative state of connected agents (neighbors) in a network. The problem of formation control aims to drive multiple agents to achieve prescribed constraints on their states [10]. In distributed approaches, the formation control is addressed by using relative measurements without relying on global coordinates. Several works have addressed the formation control for a group of nonholonomic mobile robots as a consensus problem by introducing some constant deviations for the relative states. For instance, distributed control schemes to stabilize a group of robots to achieve the desired formation in both position and orientation simultaneously are proposed in [11,12]. A control scheme that generates circular motion around virtual positions to define the desired formation is proposed in [13].

The formation tracking problem for nonholonomic robots has also been addressed using distributed schemes following the same approach based on consensus [9,14]. The aim is that the group of robots moves synchronized by tracking a reference trajectory, usually time-varying [15]. The leader-following configuration is common in the formation tracking problem, where one of the agents acts as a leader [16] or a virtual leader can be defined [17]. Due to the nonholonomic motion constraints of unicycle-type robots, dynamic feedback linearization of the kinematic model has been used to propose nonsingular control laws for the robot position, for instance, in [18]. This kind of linearization has the issue that all the robots’ linear velocities must be non-null. The forward motion of a formation has also been treated in [19] using a two-stage approach. Formation tracking of both position and orientation has been proposed to achieve asymptotic convergence in [20] or globally uniformly ultimately bounded stability in [21]. Other works, as [22,23], have proposed a state transformation to linearize the model of the robots and a distributed observer to estimate the leader’s trajectory. Then, each follower tracks the estimated trajectory using an individual controller. All the previously referred works assume that the group of robots moves in an environment without obstacles and that collisions between agents do not occur.

The capability to deal with fixed obstacles and avoid collisions is essential during the motion of a formation of nonholonomic robots [24]. A distributed collision-free formation control scheme for nonholonomic robots based on potential fields is proposed in [25]. The same problem of formation and the heading consensus is addressed in [26] using a hybrid switching control law. In general, both collision and obstacle avoidance capabilities become more important when the whole formation moves tracking the desired trajectory imposed by a leader. In the work [27], a rotational and repulsive force-based approach is proposed, which is evaluated in V-shape and circular formations. The work [28] addresses formation tracking with multiple leaders, where the team of leaders and followers are fully independent, and only the followers have collision avoidance capability. A control law with prescribed transient and steady-state performance is proposed in [29]. The collision avoidance between the followers is introduced as inequality constraints in the prescribed performance controller. These kinds of constraints are typically used in model predictive control to avoid collisions, which has also been used in formation tracking [30], with the disadvantage of the computational cost to solve the optimization problem. Other works, such as [31], focus on maintaining a rigid formation, for instance, a regular polygon, and the formation is scaled to navigate in an environment with obstacles.

The formation tracking control with obstacle avoidance can be seen as a two-task problem. Most of the described literature addresses the problem by switching between two control laws, one dedicated to the formation task and the other committed to avoiding collisions/obstacles. In [32], a multi-region control scheme for formation tracking with obstacle avoidance and connectivity preservation is proposed. In that work, a different control law is applied depending on the detection of an obstacle within concentric regions around each agent. Therefore, only one task/objective is taken into account at each time instant. In robotics, it is known that the task-function approach [33] allows the design of control laws to deal with problems involving several contradictory objectives. Although the task-function approach has been mainly used for the control of robot manipulators [34], there are examples of its application for triangular formation control of 3D vehicles [35] and formation control of unicycle-type robots [36,37,38]. Nevertheless, all the aforementioned works that have used null-space projectors to solve the formation control of nonholonomic robots are centralized control schemes relying on global measurements. We have presented preliminary results of a priority task-based distributed formation control (without tracking) to deal with obstacles for holonomic robots in the conference work [39].

In this paper, we propose to tackle the two-tasks problem of distributed formation tracking control and obstacle avoidance for unicycle-type robots, in particular differential-drive robots, in the framework of a hierarchical task-based control approach. Static feedback linearization is used to compute the robot velocities for each robot from the motion of a control point. The distributed formation control is addressed as a consensus problem of virtual agents, related to the real agents’ position by desired displacements, such that if the virtual agents achieve consensus, the real agents reach the desired formation. Obstacle avoidance becomes the task with higher priority when an obstacle is within a given security distance. An obstacle can be another agent, such that collisions between agents are avoided, or a fixed object in the environment. Since a security radius is defined for the nearest obstacle to each agent, the method assumes that the obstacles are convex and it is able to deal with polygonal obstacles. The main contributions of this work are the following:

- The proposed control scheme is fully distributed since it is based on consensus, valid for connected communication topologies, scalable for a large number of robots, and generic to define the desired formation. The computation of the robots’ velocities depends only on relative information of neighboring agents and local measurements to avoid obstacles. Neither a global coordinate system nor a map of the environment **is required. The leader is able to track any smooth time-parameterized reference, also** avoiding obstacles if needed. The leader is part of the formation, interacting with all the followers, with the advantage that the leader also adapts its motion if the followers perform avoidance actions. Existing approaches of formation tracking with obstacle avoidance are valid for specific formation shapes [27]; the leaders and followers are independent and only the followers can perform collision avoidance [28,29]; some methods focus on rigid-body formation by scaling it to avoid obstacles [31]; and some approaches are computationally costly [30].
- A task function is proposed for the distributed formation tracking, and it is combined with an obstacle avoidance task using a hierarchical scheme to solve both tasks simultaneously, which to the authors’ knowledge has not been done before in a distributed scheme. The proposed scheme guarantees continuity of the robots’ control inputs when both tasks transit to be activated or deactivated. The stability of both task function errors is proven even in the transition of tasks. Given that the control law’s design is based on the kinematics of a control point for each agent, the proposed control scheme can be easily extended for formation tracking of holonomic agents as UAVs. Existing approaches that are based on the hierarchical task-based approach tackle only the formation control (without tracking) [39] in a centralized fashion [36,37,38]. Moreover, most of the formation schemes with obstacle avoidance are discontinuous switching approaches, e.g., [24,26,32], or require a careful tuning of the weight of the different terms of both tasks [25,27].
- The proposed approach is evaluated through realistic simulations using a dynamic simulator in environments with obstacles and in real experiments with differential-drive robots. Few works on distributed formation control of nonholonomic robots have reported results in real robots, e.g., [5,13,22], but none of them solve the formation tracking with obstacle avoidance.

As a summary, we highlight some advantages of the proposed scheme with respect to different existing approaches. One classical approach is the artificial potential-based approach, which is used in [25]. With respect to that work, our approach does not require to predefine the desired position or trajectory of the whole team of robots, nor to know a map of the environment to build the potential functions. Our control law is not a direct sum of controllers for each task that might cancel in the case of conflicting tasks, but a convex combination of control laws for each task is proposed in our control scheme. Another kind of approach is the formulation of a consensus problem to solve the formation problem as in [26]. We also formulate the formation control as a consensus problem, but we extend the formulation to formation tracking where the group of robots is guided by a leader. In our case, heading consensus is naturally achieved because the group of nonholonomic robots follows a common trajectory. The main advantage of our approach is that we integrate the obstacle avoidance task using priorities and smooth transitions to avoid a hybrid switching control law, guaranteeing stability of both tasks and continuity of the robot velocities. A third kind of approach is the one based on optimization methods, as the model predictive control in [30]. Unlike that work, our approach relies on consensus using relative information between agents and is not needed to precompute the reference trajectory of each follower from the one of the leader. We provide a formal stability proof for both formation and obstacle avoidance tasks, which is not trivial in an optimization-based formulation. We also use feedback linearization of a control point, which has no singularity issues in comparison to a dynamic extension where the control law becomes singular when the robot has null translational velocity. Besides, the MPC-based approach implicitly has a computational cost to solve a quadratic programming problem in a preview horizon. In the same vein, works as [40], that uses distributed optimization for a split and merge strategy of a formation in dynamic environments, suffer from the computational burden. In that strategy, the robots compute locally optimal parameters for the formation to remain within the convex neighborhood of the robots via sequential convex programming. A low level local planner is used to generate the individual robot motion. With respect to that work, the novelty of our approach is that it directly provides the robot velocities without relying on a high level planning strategy. The control scheme presented in [41] is close to ours, however, that work addresses the rendezvous problem in environments with obstacles unlike the formation tracking that we tackle. Besides that important difference, the referred approach consists of a high level strategy that is executed relying on a low level control law and ours directly provides the robot velocities. Moreover, the novelty of our approach with respect to that method is the guarantee of continuity of the robot velocities, which is not the case of the referred work since sudden changes of orientation might occur in the robot trajectories. We consider the generation of smooth velocities important to improve the accuracy of the position control when the robots are modeled at kinematic level. Due to robot and actuator dynamics, discontinuous velocity commands cannot be exactly realized by a vehicle. Moreover, the transient to converge to the new command may lead to collisions. Sudden fast motions are not convenient in practice since the sensing capabilities of the robots might deteriorate, in our case the distance measurement might introduce noise for sudden motions.

The rest of the paper is structured as follows. Section 2 describes the model of a unicycle-type robot, and some considerations to control it. It also recalls some results on graph theory and the task-function approach. In Section 3, the problem of formation tracking for differential-drive robots with obstacle avoidance is formulated, and the proposed control scheme is detailed. Section 4 presents the performance of the proposed approach through simulations and real experiments. Finally, some concluding remarks and future work ideas are presented in Section 5.

## 2. Preliminaries

In this section, we will present the model considered for each agent of the network of robots and the basics on graph and consensus theory that are used in the control law’s design.

### 2.1. Model of the Robots

**Figure 1:**
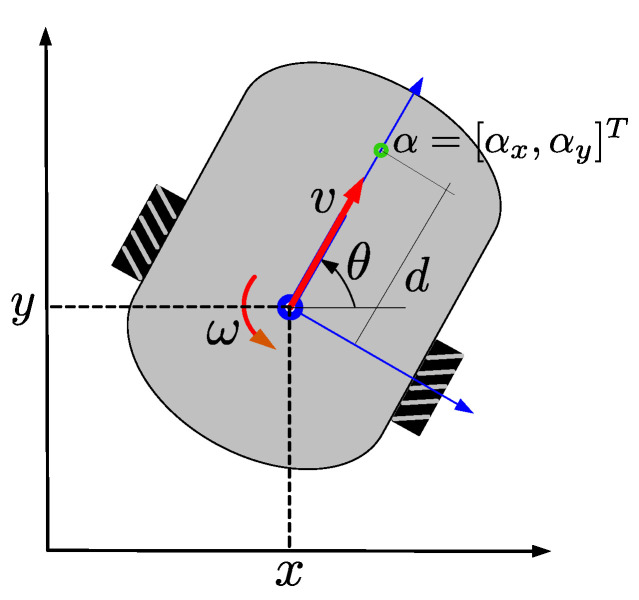
Scheme of a differential-drive mobile robot and definition of the control point α.

Let $N=\{R_{1},R_{2},\ldots ,R_{N}\}$ be a set of unicycle-type robots, in particular differential-drive robots (DDRs), moving on the xy-plane with pose $q_{i}={\left[x_{i},y_{i},\theta_{i}\right]}^{T},i\in \left\{1,\ldots ,N\right\}$. The kinematic model for each robot, according to Figure 1, is given by:

(1) $$\left[\begin{array}{c} {\dot{x}}_{i}\\ {\dot{y}}_{i}\\ {\dot{\theta }}_{i} \end{array}\right]=\left[\begin{array}{cc} \cos(\theta_{i}) & 0\\ \sin(\theta_{i}) & 0\\ 0 & 1 \end{array}\right]\left[\begin{array}{c} v_{i}\\ \omega_{i} \end{array}\right],\;i\in \left\{1,\ldots ,N\right\},$$

where $\left[x_{i},y_{i}\right]$ are the coordinates of the position of the center of rotation of the *i*-th robot, $\theta_{i}$ the orientation with respect to the *x*-axis, $v_{i}$ is the translational velocity and $\omega_{i}$ the angular velocity.

We are interested that the team of robots can achieve formation just for position, not orientation. It is known that is not possible to control the robot position $p_{i}={\left[x_{i},y_{i}\right]}^{T}$, seen as output vector, using an static feedback linearization [42]. Then, we will consider $\alpha_{i}$ as control point, which is a point out of the wheels axis as in Figure 1. For simplicity, the point $\alpha_{i}$ is chosen shifted by a distance d>0 along the longitudinal axis passing over point $p_{i}$ of the robot. The distance *d* is such that the point $\alpha_{i}$ is inside the robot periphery. The coordinates of the point $\alpha_{i}$ are given by

(2) $$\alpha_{i}=\left[\begin{array}{c} \alpha_{x_{i}}\\ \alpha_{y_{i}} \end{array}\right]=\left[\begin{array}{c} x_{i}+d\cos\left(\theta_{i}\right)\\ y_{i}+d\sin\left(\theta_{i}\right) \end{array}\right].$$

The time-derivative of the point $\alpha_{i}$ (2) is as follows

(3) $$\left[\begin{array}{c} {\dot{\alpha }}_{x_{i}}\\ {\dot{\alpha }}_{y_{i}} \end{array}\right]=M_{i}\left(\theta_{i}\right)\left[\begin{array}{c} v_{i}\\ \omega_{i} \end{array}\right],$$

where the decoupling matrix $M_{i}\left(\theta_{i}\right)$ is given by

(4) $$M_{i}\left(\theta_{i}\right)=\left[\begin{array}{cc} \cos\left(\theta_{i}\right) & -d\sin\left(\theta_{i}\right)\\ \sin\left(\theta_{i}\right) & d\cos\left(\theta_{i}\right) \end{array}\right],$$

and for each robot $R_{i}$ is not singular, since $det(M_{i}\left(\theta_{i}\right))=d\neq 0$. Then, the control point coordinates can be driven as desired by the robot velocities and the orientation angle remains as zero-dynamics of the system (1). However, it is known that such dynamics is stable when the control point is driven to a constant position or tracks a time-varying reference [42].

### 2.2. Graph and Consensus Theory

In this section, we present some notations and preliminaries about graph and consensus theory that is extensively presented, for instance, in [7,9].

In a network of agents, consensus means to reach an agreement on a certain quantity of interest that depends on the state of all agents in the network. In consensus theory, a network of agents is typically modeled as a graph G, which consists of a vertex set V(G) and an edge set E(G). An edge is denoted by ij and j∼i denotes that the vertex *i* and vertex *j* are neighbors. The neighbors of vertex *i* in a graph G is denoted by $N_{i}\left(G\right)=\left\{j:ji\in E\left(G\right)\right\}$.

The adjacency matrix $A=\left[a_{ij}\right]\in R^{N\times N}$ of a graph with *N* vertices is a square matrix with entries $a_{ij}$ corresponding to the weight of the edge ij; when *i* is not adjacent to *j* then $a_{ij}=0$. The Laplacian matrix of G is L(G)=Δ−A where $\Delta =diag(d_{1},\cdots ,d_{N})$ with $d_{i}=\sum_{j=1}^{N}a_{ij}$.

A graph G is connected if there is a path between any two vertices, otherwise it is disconnected. If the graph G is connected, then the Laplacian matrix has an eigenvalue $\lambda_{1}\left(L\right)=0$ with algebraic multiplicity one, associated to the eigenvector ${𝟙}_{N}={\left[1\;\cdots \;1\right]}^{T}$, i.e., $\ker\left(L\left(G\right)\right)=\left\{x:x_{1}=\ldots =x_{N}\right\}$. For undirected graphs the Laplacian matrix L is positive semidefinite and symmetric.

In this work, we will consider a multi-agent system (MAS) composed of *N* differential-drive robots (DDRs) modeled as in the previous section and with an associated graph G modeling the network’s connectivity. Given the expression of the dynamics to be controlled (3), it can be treated as decoupled single-integrator dynamics expressed in general form as

(5) $${\dot{x}}_{i}\left(t\right)=u_{i},\;\;i\in \left\{1,\cdots ,N\right\},$$

where $x_{i},u_{i}\left(t\right)\in R^{2}$ are the state and auxiliary control input of agent *i*, respectively. The network dynamics for the agents (5) can be written in vector form as

(6) $$\dot{x}\left(t\right)=u\left(t\right),$$

with $x\left(t\right)={\left[x_{1}{\left(t\right)}^{T},\cdots ,x_{N}{\left(t\right)}^{T}\right]}^{T}\in R^{2N}$ and $u\left(t\right)={\left[u_{1}{\left(t\right)}^{T},\cdots ,u_{N}{\left(t\right)}^{T}\right]}^{T}\in R^{2N}$.

According to [7], the consensus error for agent *i* with respect to its neighbors is defined as

(7) $$e_{c_{i}}\left(t\right)=\sum_{j\in N_{i}}a_{ij}\left(x_{j}\left(t\right)-x_{i}\left(t\right)\right),\;\;i\in \left\{1,\cdots ,N\right\}.$$

If this error is zero for all the agents, it is accomplished that $x_{i}=x_{j}$ for all *i*,j∈E(G), i≠j. The individual consensus error (7) can be expressed in matrix form as

(8) $$e_{c}\left(t\right)={\left[e_{c_{1}}{\left(t\right)}^{T},\cdots ,e_{c_{N}}{\left(t\right)}^{T}\right]}^{T}=-\left(L⊗I_{2}\right)x\left(t\right)\in R^{2N},$$

where ⊗ denotes the Kronecker product and $I_{2}$ is the identity matrix of size 2.

### 2.3. Hierarchical Task-Based Control Approach

Let us consider one agent of the form (5) with state $q\in R^{n}$ and a differential mapping, denoted by $X\left(q\right)\in R^{m}$, between a task space and the state space, which models a task to be performed by the agent. When addressing a regulation problem not a tracking one, it is desired that the task reaches a constant value $X^{d}$ by enforcing the convergence to zero of the error function

(9) $$e\left(q\right)=X\left(q\right)-X^{d}\in R^{m}.$$

The time-derivative of (9) is given by

(10) $$\dot{e}=J\left(q\right)\dot{q},$$

being $J\left(q\right)\in R^{m\times n}$ a Jacobian matrix. In (10), $\dot{q}$ can be seen as the vector of control inputs, and to find it out, the Moore–Penrose pseudoinverse of the Jacobian matrix can be used as follows

(11) $$\dot{q}=J^{+}\left(q\right)\dot{e},$$

with $J^{+}\left(q\right)=J^{T}\left(q\right){\left(J\left(q\right)J^{T}\left(q\right)\right)}^{-1}\in R^{n\times m}$. The task space control approach is valid if the dimension of the task space is less than or equal to the dimension of the state space of the agent m≤n [43]. The desired dynamics of the task error function is as follows

(12) $$\dot{e}=-\lambda e,$$

with λ>0, such that exponential convergence of the task function to its desired value is guaranteed by using the vector of control inputs

(13) $$\dot{q}=-\lambda J^{+}\left(q\right)e\left(q\right).$$

Since we address the problem that a group of DDRs reaches a desired formation avoiding collisions/obstacles when needed, two tasks must be carried out simultaneously. The hierarchical task-based approach introduced in [33] allows two tasks to be solved as good as possible, in the sense that the solution of the task with higher priority is guaranteed and the solution of the task with lower priority is subject to the former [36,44,45].

The hierarchical task-space approach requires the formulation of some projectors. Following [44], the null space projector of the *i*-th task, considered the priority task, is computed as

(14) $$N_{i}=I_{n}-J_{i}^{+}\left(q\right)J_{i}\left(q\right)\in R^{n\times n}.$$

As an example, consider two tasks $X_{1}$ and $X_{2}$, where $X_{1}$ has the highest priority. According [36,44], the total control action is given by

(15) $$\dot{q}={\dot{q}}_{1}+N_{1}{\dot{q}}_{2},$$

where ${\dot{q}}_{1}=-\lambda_{1}J_{1}^{+}\left(q\right)e_{1}\left(q\right)\in R^{n}$ is the input computed individually from $X_{1}$ and ${\dot{q}}_{2}=-\lambda_{2}J_{2}^{+}\left(q\right)e_{2}\left(q\right)\in R^{n}$ is the input obtained from $X_{2}$.

It is common that initially a system only has to solve one task by using the control inputs vector (13), and in some moment the system requires to solve more than one task. Then, in the case of two tasks the control inputs vector must switch from (13) to (15) and eventually the system returns to solve only the initial task.

## 3. Problem Formulation and Proposed Solution

Let N be a set of DDRs as described in Section 2.1. Consider that there exists a finite set of obstacles in the environment. The agents themselves can be obstacles for the others or there may be fixed obstacles in the environment.

The problem addressed in this work is to design a control law for each agent $u_{i}=\gamma \left(\alpha_{i},N_{i}\right)$, i={1,…,N} to achieve:

- Asymptotic tracking of a predefined smooth trajectory m(t) by the leader, for instance, considered as the robot $R_{1}$
  (16) $$\lim_{t\to \infty }\left(\alpha_{1}\left(t\right)-m\left(t\right)\right)=0,$$
  where $\alpha_{1}\left(t\right)$ is the position of the control point of the leader.
- Asymptotic convergence to a desired formation by the follower agents, i.e.,
  (17) $$\lim_{t\to \infty }a_{ij}\left(\alpha_{i}\left(t\right)-\alpha_{j}\left(t\right)\right)=\Delta_{ij}=const.,\;i,j\in \left\{1,\ldots ,N\right\},i\neq j,$$
  where $\Delta_{ij}$ denotes desired constant offsets between agents to make that the desired formation has a fixed-predefined form.
- Obstacle avoidance between agents and with respect to any fixed object in the environment, always maintaining a safety distance σ, i.e., the condition
  (18) $$∥\alpha_{i}-\alpha_{o}^{i}∥>\sigma$$
  must be accomplished, where $\alpha_{o}^{i}$ is the position of the nearest obstacle to the *i*-th agent.

To solve the enunciated problem, the following assumptions are considered:

- Each robot has omnidirectional capability to measure distance and only the nearest obstacle is considered to avoid it at a time.
- The obstacles are considered convex since a circumference that must not be trespassed is defined around the nearest point on an obstacle from each robot.
- The communication topology for the group of robots is considered connected.
- The robots velocities are available for their neighbors and they are communicated according to the communication topology.

The described problem requires to solve two tasks, the formation tracking and the obstacle avoidance. We will use the hierarchical task-based formulation to guarantee that both tasks are adequately accomplished. Most of the time, the formation tracking task is active, but both task must be solved simultaneously when obstacles are close to an agent. The formulation of each task is presented in the following sections.

### 3.1. Task of Obstacle Avoidance

Every robot $R_{i}$ must avoid collisions with obstacles (either fixed obstacles or other agents). This task is activated when $∥\alpha_{i}-\alpha_{o}^{i}∥\leq \sigma$, such that the agents must always maintain a safety distance σ to the obstacles and has priority over the formation tracking. To meet this objective, the task $x_{1}^{i}=\rho \left(\alpha_{i}\right)$ is defined as the relative distance from the robot position $\alpha_{i}$ to the position $\alpha_{o}$ of the nearest obstacle as

(19) $$\rho \left(\alpha_{i}\right)=∥\alpha_{i}-\alpha_{o}^{i}∥\in R.$$

Given the assumption of sensing capabilities of the robots, the distance $\rho (\alpha_{i})$ can be measured by each robot $R_{i}$ and thus, the global position of the obstacle is not required. Thus, the following error function is defined

(20) $$e_{o_{i}}=\rho \left(\alpha_{i}\right)-\sigma \in R.$$

The dynamics of the error is given by:

(21) $${\dot{e}}_{o_{i}}=J_{o_{i}}\left(\alpha_{i}\right){\dot{\alpha }}_{i},$$

where

(22) $$J_{o_{i}}\left(\alpha_{i}\right)=\frac{{\left(\alpha_{i}-\alpha_{o}^{i}\right)}^{T}}{\rho (\alpha_{i})}\in R^{1\times 2}.$$

As desired dynamics for (21) we want to have

(23) $${\dot{e}}_{o_{i}}=-\lambda e_{o_{i}},$$

with λ>0. Solving for $\alpha_{i}$, the feedback control law for obstacle avoidance is given by

(24) $${\dot{\alpha }}_{i}=-\lambda J_{o_{i}}^{+}\left(\alpha_{i}\right)e_{o_{i}},$$

where $J_{o_{i}}^{+}\left(\alpha_{i}\right)=J_{o_{i}}^{T}\left(\alpha_{i}\right){\left(J_{o_{i}}\left(\alpha_{i}\right)J_{o_{i}}^{T}\left(\alpha_{i}\right)\right)}^{-1}$.

### 3.2. Task of Agents’ Formation

The formation control problem is addressed in this work as a consensus problem in order to achieve a distributed and scalable formulation. Consider a set of *N* DDRs connected through a communication network such that each one exchanges information with a set of neighbors. We will consider dynamics for each DDR as in (3)

(25) $${\dot{\alpha }}_{i}=M_{i}\left(\theta_{i}\right)u_{i}\left(t\right),\;i\in \left\{1,\ldots ,N\right\}.$$

Agents’ formation can be achieved by consensus of a virtual network [26]. We will specify the desired formation as a set of fixed translation vectors $z_{i}\in R^{2}$ with respect to an arbitrary common reference frame, thus the position $\alpha_{i}$ of the *i*-th agent is related to the position $\alpha_{v_{i}}$ of the virtual agent by

(26) $$\alpha_{v_{i}}=\alpha_{i}+z_{i},\in R^{2},\;i\in \left\{1,\ldots ,N\right\},$$

such that if the virtual agents reach the same value, i.e., $\alpha_{v_{i}}^{*}=\alpha_{v}^{*}\in R^{2},i\in \left\{1,\ldots ,N\right\}$, then the real agents will reach the desired formation given by the vectors $z_{i}$. Notice that the network of virtual agents has the same Laplacian matrix than the original network and the virtual agent’s dynamics is given by

(27) $${\dot{\alpha }}_{v_{i}}={\dot{\alpha }}_{i}=M_{i}\left(\theta_{i}\right)u_{i}\left(t\right).$$

The consensus error for the virtual agent *i* with respect to its neighbors is

(28) $$e_{v_{i}}=\sum_{j\in N_{i}}\left(a_{ij}\left(\alpha_{v_{j}}-\alpha_{v_{i}}\right)\right)\in R^{2},$$

and the consensus error vector is

(29) $$e_{v}={\left[e_{v_{1}}^{T},e_{v_{2}}^{T},\ldots ,e_{v_{N}}^{T}\right]}^{T}=-\left(L⊗I_{2}\right)\alpha_{v}\in R^{2N},$$

with $\alpha_{v}={\left[\alpha_{v_{1}}^{T},\alpha_{v_{2}}^{T},\ldots ,\alpha_{v_{N}}^{T}\right]}^{T}$. Applying control inputs of the form $u_{i}\left(t\right)=M_{i}^{-1}\left(\theta_{i}\right)e_{v_{i}}$ to the systems (25), the virtual agents will reach consensus and consequently the real agents will reach the desired formation specified by the vectors $z_{i}$.

As consensus can be reached for the virtual agents’ state, such that each virtual agent reaches the position $\alpha_{v}^{*}$, this allows us to formulate a secondary task $x_{2}^{i}=\alpha_{v_{i}}$ in the hierarchical task-based approach to solve the formation control with obstacle avoidance. Thus, the error of the consensus task (29) can be rewritten as

(30) $$e_{v}=\alpha_{v}-\left({𝟙}_{N}⊗\alpha_{v}^{*}\right)\in R^{2N}.$$

Thus, the corresponding time-derivative of the consensus task is given by

(31) $${\dot{e}}_{v}=J_{v}\left(\alpha_{v}\right){\dot{\alpha }}_{v},$$

where $J_{v}\left(\alpha_{v}\right)=I_{N}⊗J_{v_{i}}\left(\alpha_{v_{i}}\right)$. Since $J_{v_{i}}\left(\alpha_{v_{i}}\right)=I_{2}$, then the whole Jacobian matrix of the consensus task is $J_{v}\left(\alpha_{v}\right)=I_{2N}\in R^{2N\times 2N}$. In the sequel, we will not denote the dependence of the Jacobians from $\alpha_{v}$. In the following sections, we will use this formulation of consensus of virtual agents to propose a formation tracking control law for a group of DDRs.

#### Formation Tracking Control Law

Let us address the problem where a leader robot $R_{1}$ have to track a predefined time-varying trajectory m(t) and the followers must follow the leader in formation (equivalently the virtual agents follow the leader in consensus), first assuming that no avoidance actions are required. The following distributed control protocol is proposed to solve this problem:

(32) $$\begin{array}{cc} u_{1} & =M_{1}^{-1}\left(\theta_{1}\right)\left[-\gamma \left(\alpha_{1}-m\right)+\dot{m}-k\sum_{j\in N_{1}}a_{1j}\left(\alpha_{v_{1}}-\alpha_{v_{j}}\right)\right], \end{array}$$

(33) $$\begin{array}{cc} u_{i} & =M_{i}^{-1}\left(\theta_{i}\right)\left\{\frac{1}{|N_{i}|}\sum_{j\in N_{i}}a_{ij}\left[{\dot{\alpha }}_{j}-k\left(\alpha_{v_{i}}-\alpha_{v_{j}}\right)\right]\right\},\;i\in \left\{2,\ldots ,N\right\}, \end{array}$$

where $M_{i}^{-1}\left(\theta_{i}\right)$ is the inverse of the decoupling matrix associated to each robot, $\dot{m}$ is the velocity of the desired trajectory, γ is the tracking control gain and *k* is the formation control gain.

The vector of position error for the whole MAS, seen as a new task function error, is given by

(34) $$e_{r}=\alpha_{v}-r\in R^{2N},$$

where $r={\left[{\left(m+z_{1}\right)}^{T},\frac{1}{|N_{2}|}\sum_{j\in N_{2}}a_{2j}\alpha_{v_{j}}^{T},\frac{1}{|N_{3}|}\sum_{j\in N_{3}}a_{3j}\alpha_{v_{j}}^{T},\ldots ,\frac{1}{|N_{N}|}\sum_{j\in N_{N}}a_{Nj}\alpha_{v_{j}}^{T}\right]}^{T}$ and $z_{1}$ is the relative constant displacement vector assigned to the leader in the formation. The dynamics of the error (34) are given by

(35) $${\dot{e}}_{r}=J_{r}{\dot{\alpha }}_{v}-\dot{r}\in R^{2N},$$

with $\dot{r}={\left[{\dot{m}}^{T},\frac{1}{|N_{2}|}\sum_{j\in N_{2}}a_{2j}{\dot{\alpha }}_{v_{j}}^{T},\frac{1}{|N_{3}|}\sum_{j\in N_{3}}a_{3j}{\dot{\alpha }}_{v_{j}}^{T},\ldots ,\frac{1}{|N_{N}|}\sum_{j\in N_{N}}a_{Nj}{\dot{\alpha }}_{v_{j}}^{T}\right]}^{T}$ and $J_{r}=I_{2N}$, where for each agent $J_{r_{i}}=I_{2}$.

Since the velocities of the virtual agents depend directly on the control input and the decoupling matrix, we rewrite (35) as

(36) $${\dot{e}}_{r}=M\left(\theta \right)u-\dot{r},$$

with $u={\left[u_{1}^{T},u_{2}^{T},\ldots ,u_{N}^{T}\right]}^{T}$ and $M\left(\theta \right)=diag(M_{1}\left(\theta_{1}\right),\ldots ,M_{N}\left(\theta_{N}\right))$.

**Theorem** **1.** *Consider an MAS of N unicycle-type robots with position dynamics of the control point as in* (3), *under a connected communication graph G, and related to the virtual agents’ position according to* (26). *The leader robot knows a continuously differentiable reference trajectory m(t). Then, for control parameters γ>0 and k>0 of the distributed protocol* (32) *and* (33)*, each component of the error function $e_{r}$* (34) *convergences asymptotically to zero. Therefore, the leader’s position converges to the desired trajectory and the followers converge to the specified formation around the leader.*

**Proof.**  First, we rewrite the control inputs (32) and (33) in terms of the errors as follows

- For the leader
  $$\begin{array}{cc} u_{1} & =M_{1}^{-1}\left(\theta_{1}\right)\left[-\gamma e_{r_{1}}+\dot{m}-k\sum_{j\in N_{1}}a_{1j}\left(\alpha_{v_{1}}-\alpha_{v_{j}}\right)\right]. \end{array}$$
- For the followers
  $$\begin{array}{cc} u_{i} & =M_{i}^{-1}\left(\theta_{i}\right)\left[\frac{1}{|N_{i}|}\sum_{j\in N_{i}}a_{ij}{\dot{\alpha }}_{v_{j}}-ke_{r_{i}}\right]\;i\in \left\{2,\ldots ,N\right\}. \end{array}$$

Then, the control inputs in vector form are given by

(37) $$u=M^{-1}\left(\theta \right)\left[-Ke_{r}+\dot{r}-d\right],$$

where $K=\mathrm{diag}\left(\gamma ,\gamma ,k,k,k,\ldots ,k\right)\in R^{2N\times 2N}$ and $d={\left[k\sum_{j\in N_{1}}a_{1j}{\left(\alpha_{v_{1}}-\alpha_{v_{j}}\right)}^{T},0,0,\ldots ,0\right]}^{T}\in R^{2N}$.

By introducing the control input (37) in the dynamics of the system errors (36) to get the closed loop dynamics, it is obtained

(38) $${\dot{e}}_{r}=-Ke_{r}-d.$$

By expanding (38) we obtain the error dynamics of the leader and the followers

(39) $$\begin{array}{cc} {\dot{e}}_{r_{1}} & =-\gamma e_{r_{1}}+k\sum_{j\in N_{1}}a_{1j}\left(\alpha_{v_{j}}-\alpha_{v_{1}}\right), \end{array}$$

(40) $$\begin{array}{cc} {\dot{e}}_{r_{i}} & =-ke_{r_{i}}\;i\in \left\{2,\ldots ,N\right\}. \end{array}$$

On the one hand, the second term of the leader’s error dynamics (39) is the consensus error of the leader with respect to its neighbor followers, i.e., $e_{v_{1}}$ of (29). On the other hand, the dynamics of () that represents the consensus errors for the agents {2,…,N} clearly have asymptotic stability to zero. Then, according (34) and (29), at steady state we have

(41) $$e_{r_{i}}=e_{v_{i}}=\alpha_{v_{i}}-\frac{1}{|N_{i}|}\sum_{j\in N_{i}}a_{ij}\left(\alpha_{v_{j}}\right)=0\;i\in \left\{2,\ldots ,N\right\}.$$

Since $e_{v_{1}}$ depends on the first row of the Laplacian matrix as given by (29) and that row is linearly dependent on the rest of rows of the matrix, $e_{v_{i}}=0$ for i∈{2,…,N} implies that $e_{v_{1}}=0$. Therefore, the second term of (39) vanishes as the time elapses and also the tracking error $e_{r_{1}}$ converges asymptotically to zero. Then the virtual followers achieve consensus, and consequently, the real robots achieve the desired formation defined by the displacement vectors $z_{i}$ according (26), tracking the leader in formation. □

The previous theorem demonstrates the stability of the multi-agent system in closed loop with the control protocol that solves the formation tracking task. In the next section, this result will be integrated into a protocol that also includes the obstacle avoidance task.

### 3.3. Hierarchical Combination of Formation Tracking and Obstacle Avoidance Tasks

The problem formulated in Section 3 can be addressed by following the task-based approach described in Section 2.3. When an obstacle is detected, an avoidance action must be performed with higher priority over the formation tracking, but taking into account both tasks simultaneously. However, the direct switching between the formation tracking control law ((32) and (33)) and a control law combining both tasks generates discontinuous robot velocities. To alleviate this issue, we propose the use of a smooth transition as presented in [45]. Then, the velocities for each robot that combines the obstacle avoidance task, denoted by subscript *o*, as primary task, and DDRs formation tracking, denoted by subscript *r*, as secondary task is given by

(42) $$u_{i}=M_{i}^{-1}\left(\theta_{i}\right)\left({\dot{\alpha }}_{o_{i}}^{\prime }+{\dot{\alpha }}_{{r|o}_{i}}\right),\;i\in \left\{1,\ldots ,N\right\},$$

where

$$\begin{array}{cc} {\dot{\alpha }}_{o_{i}}^{\prime } & =J_{o_{i}}^{+}{\dot{e}}_{o_{i}}^{\prime },\\ {\dot{\alpha }}_{{r|o}_{i}} & ={\left(J_{r_{i}}N_{o_{i}}\right)}^{+}\left({\dot{e}}_{r_{i}}+{\dot{r}}_{i}-J_{r_{i}}J_{o_{i}}^{+}{\dot{e}}_{o_{i}}^{\prime }\right),\\ {\dot{e}}_{o_{i}}^{\prime } & =h\left(t\right){\dot{e}}_{o_{i}}+\left(1-h\left(t\right)\right)J_{o_{i}}J_{r_{i}}^{+}{\dot{e}}_{r_{i}}, \end{array}$$

with $N_{o_{i}}=I_{2}-J_{o_{i}}^{+}J_{o_{i}}\in R^{2\times 2}$, $J_{o_{i}}$ as in (22) and $J_{r_{i}}=I_{2}$ as stated below of (35), ${\dot{e}}_{o_{i}}$ as in (23) and ${\dot{e}}_{r_{i}}$ as in (39) and (40). The transition function h(t) is a smooth bounded function such that 0≤h(t)≤1 and increases from 0 to 1 (activated) when an obstacle is detected within the safety distance σ. Its value is h(t)=1 while the obstacle is within the safety distance and decreases from 1 to 0 (deactivated) when the obstacle leaves the safety distance. The duration of the transition function is fixed by the user and is the same value for both activation and deactivation. Thus, the use of the transition function yields continuity of the computed robots velocities. In the sequel we will denote h(t)=h.

In the following theorem, the stability of the control law for both obstacle avoidance and formation tracking tasks is proven. All the expressions in the theorem are presented in vector form, however, the approach keeps distributed as given by the individual control law (42) for each agent.

**Theorem** **2.** *Consider an MAS of N unicycle-type robots with position dynamics of the control point as in* (3), *under a connected communication graph G, and related to the virtual agents’ position according to* (26). *The leader robot knows a continuously differentiable reference trajectory m(t). Then, for control parameters γ>0, k>0 and λ>0 of the distributed protocol expressed in vector form*

(43) $$u=M^{-1}\left(\theta \right)\left({\dot{\alpha }}_{o}^{\prime }+{\dot{\alpha }}_{r|o}\right),$$

*where ${\dot{\alpha }}_{o}^{\prime }=J_{o}^{+}{\dot{e}}_{o}^{\prime }$, ${\dot{\alpha }}_{r|o}={\left(J_{r}N_{o}\right)}^{+}\left({\dot{e}}_{r}+\dot{r}-J_{r}J_{o}^{+}{\dot{e}}_{o}^{\prime }\right)$, ${\dot{e}}_{o}^{\prime }=h{\dot{e}}_{o}+\left(1-h\right)J_{o}J_{r}^{+}{\dot{e}}_{r}$, both the formation tracking error $e_{r}$ and the obstacle avoidance error $e_{o}$ convergence to zero asymptotically when both tasks are active, i.e., with 0<h≤1. The terms of* (43) *are as follows: ${\dot{e}}_{r}$ given by* (38), ${\dot{e}}_{o}=-\lambda e_{o}\in R^{N}$, with $e_{o}={\left[e_{o_{1}},e_{o_{2}},\ldots ,e_{o_{N}}\right]}^{T}$, ${\left(J_{r}N_{o}\right)}^{+}=diag\left({\left(J_{r_{1}}N_{o_{1}}\right)}^{+},\ldots ,{\left(J_{r_{N}}N_{o_{N}}\right)}^{+}\right)$, $J_{o}^{+}=diag\left(J_{o_{1}}^{+},\ldots ,J_{o_{N}}^{+}\right)$, $N_{o}=diag\left(N_{o_{1}},\ldots ,N_{o_{N}}\right)$, *where the individual entries have been defined below* (42).

**Proof.**  Let us consider the following extended error function

(44) $$e^{\prime }=\left[\begin{array}{c} e_{r}\\ e_{o} \end{array}\right].$$

Consider a Lyapunov function candidate given by

(45) $$V=\frac{1}{2}{e^{\prime }}^{T}e^{\prime },$$

whose time-derivative is given by

(46) $$\dot{V}={e^{\prime }}^{T}{\dot{e}}^{\prime }.$$

Using the open-loop dynamics of ${\dot{e}}_{r}$ and ${\dot{e}}_{o}$ for the whole network to compute $\dot{V}$, we have the former given by (35). Expanding the obstacle task error for the *N* agents, we get

(47) $${\dot{e}}_{o}=J_{o}\dot{\alpha }=diag\left(J_{o_{1}},\ldots ,J_{o_{N}}\right)\dot{\alpha },$$

where $J_{o}\in R^{N\times nN}$. Now, we have

(48) $$\dot{V}=\left[\begin{array}{cc} e_{r}^{T} & e_{o}^{T} \end{array}\right]\left(\left[\begin{array}{c} J_{r}\\ J_{o} \end{array}\right]{\dot{\alpha }}_{v}-\left[\begin{array}{c} \dot{r}\\ 0 \end{array}\right]\right).$$

Considering that ${\dot{\alpha }}_{v}=M\left(\theta \right)u$ according to (27) and introducing the hierarchical task-based control protocol (43), then

(49) $$\dot{V}=\left[\begin{array}{cc} e_{r}^{T} & e_{o}^{T} \end{array}\right]\left(\Psi -\left[\begin{array}{c} \dot{r}\\ 0 \end{array}\right]\right),$$

where

(50) $$\Psi =\left[\begin{array}{c} \Psi_{1}\\ \Psi_{2} \end{array}\right]=\left[\begin{array}{c} J_{r}\left(J_{o}^{+}{\dot{e}}_{o}^{\prime }\right)+J_{r}\left({\left(J_{r}N_{o}\right)}^{+}\left({\dot{e}}_{r}+\dot{r}-J_{r}J_{o}^{+}{\dot{e}}_{o}^{\prime }\right)\right)\\ J_{o}\left(J_{o}^{+}{\dot{e}}_{o}^{\prime }\right)+J_{o}\left({\left(J_{r}N_{o}\right)}^{+}\left({\dot{e}}_{r}+\dot{r}-J_{r}J_{o}^{+}{\dot{e}}_{o}^{\prime }\right)\right) \end{array}\right].$$

Expanding the expressions using ${\dot{e}}_{o}^{\prime }$ as in (43), we have

$$\begin{array}{cc} \Psi_{1} & =J_{r}J_{o}^{+}\left[h{\dot{e}}_{o}+\left(1-h\right)J_{o}J_{r}^{+}{\dot{e}}_{r}\right]+J_{r}{\left(J_{r}N_{o}\right)}^{+}\left\{{\dot{e}}_{r}+\dot{r}-J_{r}J_{o}^{+}\left[h{\dot{e}}_{o}+\left(1-h\right)J_{o}J_{r}^{+}{\dot{e}}_{r}\right]\right\},\\ \Psi_{2} & =J_{o}J_{o}^{+}\left[h{\dot{e}}_{o}+\left(1-h\right)J_{o}J_{r}^{+}{\dot{e}}_{r}\right]+J_{o}{\left(J_{r}N_{o}\right)}^{+}\left\{{\dot{e}}_{r}+\dot{r}-J_{r}J_{o}^{+}\left[h{\dot{e}}_{o}+\left(1-h\right)J_{o}J_{r}^{+}{\dot{e}}_{r}\right]\right\}. \end{array}$$

Given the known properties $J_{o}J_{o}^{+}=I_{N}$, $J_{o}N_{o}=0_{N\times 2N}$, $N_{o}=N_{o}^{T}$, and $N_{o}N_{o}=N_{o}$, the following holds

(51) $$\begin{array}{cc} J_{o}{\left(J_{r}N_{o}\right)}^{+} & =J_{o}N_{o}^{T}{\left(N_{o}N_{o}^{T}\right)}^{-1}=J_{o}N_{o}{\left(N_{o}N_{o}^{T}\right)}^{-1}=0_{N\times 2N}, \end{array}$$

(52) $$\begin{array}{cc} {\left(J_{r}N_{o}\right)}^{+} & ={\left(J_{r}N_{o}\right)}^{T}{\left(\left(J_{r}N_{o}\right){\left(J_{r}N_{o}\right)}^{T}\right)}^{-1}=N_{o}{\left(N_{o}N_{o}^{T}\right)}^{-1}=I_{2N}. \end{array}$$

Thus, $\Psi_{1}$ and $\Psi_{2}$ are simplified as follows

(53) $$\Psi =\left[\begin{array}{c} \Psi_{1}\\ \Psi_{2} \end{array}\right]=\left[\begin{array}{c} {\dot{e}}_{r}+\dot{r}\\ \left(1-h\right)J_{o}{\dot{e}}_{r}+h{\dot{e}}_{o} \end{array}\right].$$

Introducing the closed-loop dynamics (38) and ${\dot{e}}_{o}=-\lambda e_{o}$ in (53), the time-derivative of the Lyapunov function, is given by

(54) $$\dot{V}=-\left[\begin{array}{cc} e_{r}^{T} & e_{o}^{T} \end{array}\right]\left(P\left[\begin{array}{c} e_{r}\\ e_{o} \end{array}\right]-\left[\begin{array}{c} d\\ 0 \end{array}\right]\right),$$

where

(55) $$P=\left[\begin{array}{cc} \left(K⊗I_{2}\right) & 0_{2N\times N}\\ \left(1-h\right)J_{o}\left(K⊗I_{2}\right) & \lambda hI_{N} \end{array}\right].$$

The eigenvalues of matrix P depend on the values λ>0, 0<h(t)≤1 and the matrix K, which is defined as $K=\mathrm{diag}\left(\gamma ,\gamma ,k,k,k,\ldots ,k\right)\in R^{2N\times 2N}$, where γ>0 is the tracking gain and k>0 is the consensus gain. Therefore, the matrix P is positive definite. As in the case without obstacle avoidance of the Theorem 1, d only affects the dynamics of the tracking error $e_{r_{1}}$ and represents the consensus error of the leader with respect to its neighbors. Given the positive definiteness of the matrix P, the rest of the consensus errors $e_{r_{i}}$, i∈{2,…,N} altogether with the evasion error $e_{o}$ have asymptotic stability to zero. As shown below (41), the effect of d vanishes as the time elapses and the tracking error also converges asymptotically to zero. Consequently, the leader tracks the time-varying reference, the virtual followers reach consensus to the leader position (respectively the robots achieve the desired formation around the leader) and the robots perform obstacle avoidance when needed. □

The previous theorem guarantees the stability of the multi-agent system in closed loop with the distributed control protocol that solves both the formation tracking and obstacle avoidance tasks and provides continuity of the computed robot velocities. Since we are modeling the robots at kinematic level, the generation of smooth velocities is important to improve the accuracy of the position control. It is well known that due to robot and actuator dynamics (masses and rotational inertias), velocity commands with discontinuous profile cannot be exactly realized by a vehicle.

From our point of view, the proposed approach has two limitations. (1) Connectivity of the communication model is very important to guarantee convergence of the consensus error; however, connectivity can be easily lost when the robots move in cluttered environments. Although our scheme does not consider an explicit handling of connectivity, the consensus control law favors the maintenance of it. Nevertheless, there are no theoretical guarantees that connectivity is preserved in the current formulation. We consider that the approach can be extended to include an additional task dedicated to contribute in the velocities’ computation to move the group of robots preserving connectivity, such that the control law might be a combination of the solution of three tasks. (2) The proposed approach is considered as a sensor-based reactive navigation method that works properly not only for circular (strongly convex) obstacles but also for unknown convex polygonal obstacles of different forms. Nevertheless, our approach is not able to deal with non-convex obstacles. We consider that the proposed approach offers a good compromise between effectiveness and required information to solve the formation tracking problem in cluttered environments without the need of a high level global planner.

## 4. Evaluation of the Proposed Scheme

In this section, we present results of implementing the proposed distributed control law, first in the dynamic simulator Gazebo with a group of TurtleBots 3 Burger (Robotis Inc., Lake Forest, CA, USA) and then, with real robots using the Robot Operating System (ROS) in both cases. The simulations are presented for two different cases: a 4-agent system where the predefined trajectory to follow was a quadrifolium curve and a 10-agent system that had to follow a circular predefined trajectory. Results in an environment with unknown polygonal obstacles are also shown.

In the implementation, the distance *d* to define the control point (2) for all the robots was set to 10 cm. Besides, the transition function is $h\left(t\right)=\frac{1}{2}\left(1-\cos\left(\pi \frac{\left(t-t_{0}\right)}{\left(t_{f}-t_{0}\right)}\right)\right)$, where $t_{0}$ was set to the current time value ($t_{0}=t$) at the instant that a DDR crossed the security distance of an obstacle, $t_{f}=t_{0}+t_{d}$ with $t_{d}$ the duration of the transition function that was set to 0.8 s for both activation and deactivation. The sampling time in the simulations was set to 25 ms. Since the formation tracking control law requires knowing the velocities of the neighboring robots in the formation, we used estimated values taken from the velocities computed by the control law in one sampling time before the current time. This aspect could be improved as future work by using, for instance, an estimation of the velocities given by an exact differentiator [46] of the robots positions, or a distributed observer as in [47], although in the last case only estimation of the leader’s state would be needed.

### 4.1. A 4-Agent System

**Figure 2:**
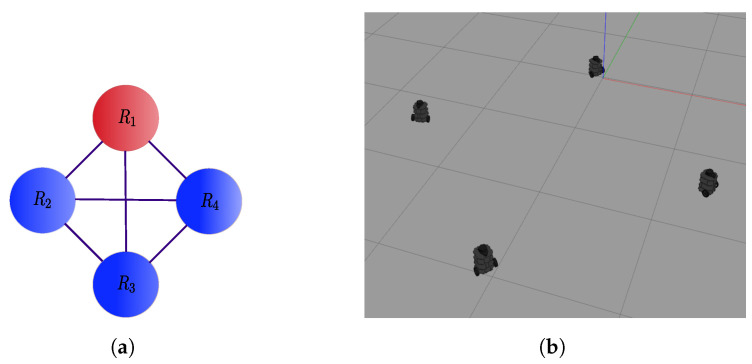
The 4-agent system. (**a**) Connected graph used in the simulations. (**b**) Specified formation in the dynamic simulator Gazebo.

The results to implement the distributed control law (42) for a 4-agent system (N=4) are presented in this section. We considered the network with undirected communication topology described by the graph of Figure 2a and the desired formation of Figure 2b. We set $a_{ij}=1$ for connected agents and initial conditions $q_{x}\left(0\right)={\left[0,-2,-3,-1\right]}^{T}$, $q_{y}\left(0\right)={\left[0,1.8,-1,-0.8\right]}^{T}$ and $q_{\theta }\left(0\right)={\left[0,-0.6,1.14,0\right]}^{T}$ for agents 1 to 4, respectively. The reference trajectory had a duration of τ=200 s and was given by $m\left(t\right)={4\left[\sin(4\pi t/\tau \right)\cos2\pi t/\tau ,\sin(4\pi t/\tau )\sin2\pi t/\tau ]}^{T}$. There was a fixed obstacle in the environment, in particular a column in the position $\alpha_{o}={\left[0,-2\right]}^{T}$, and the security distance for it and between robots was set to σ=0.6 m. The displacement vectors to define the desired formation were $z_{1}=1.5{\left[\cos\left(3\pi /2\right),\sin\left(3\pi /2\right)\right]}^{T}$, $z_{2}=1.5{\left[\cos\left(2\pi \right),\sin\left(2\pi \right)\right]}^{T}$, $z_{3}=1.5{\left[\cos\left(\pi /2\right),\sin\left(\pi /2\right)\right]}^{T}$ and $z_{4}=1.5{\left[\cos\left(\pi \right),\sin\left(\pi \right)\right]}^{T}$, for agents $R_{1}$ to $R_{4}$ respectively, with respect to the formation center. The control gains were set to γ=1, λ=0.8 and k=0.1 for tracking, evasion and consensus, respectively.

**Figure 3:**
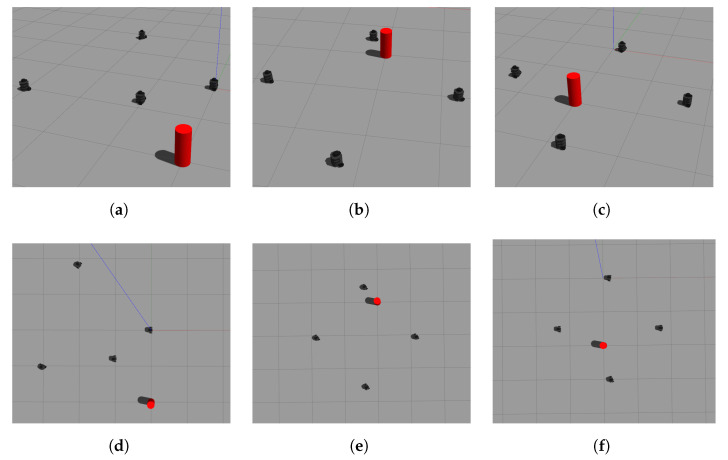
The 4-agent system. Snapshots of the DDRs motion in the simulation. (**a**) Initial position. (**b**) Intermediate position. (**c**) Final position. (**d**) Upper view of initial position. (**e**) Upper view of intermediate position. (**f**) Upper view of final position.

**Figure 4:**
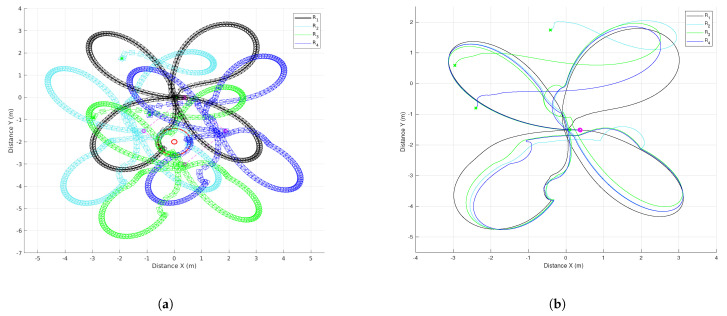
The 4-agent system. Trajectories of the DDRs. (**a**) Trajectories of the real agents. (**b**). Trajectories of the virtual agents.

**Figure 5:**
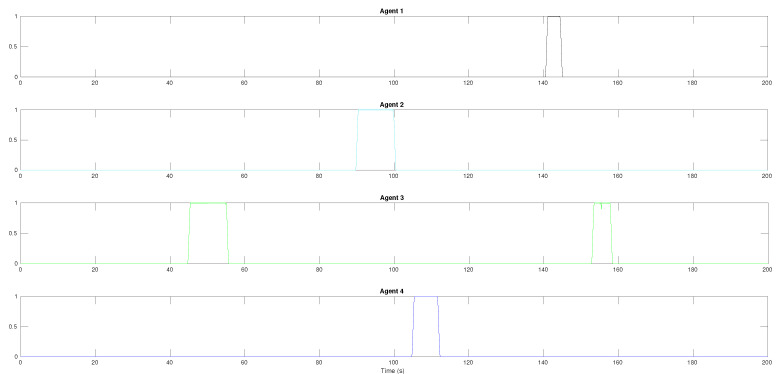
The 4-agent system. Task transition functions for each agent.

The results of the formation tracking with obstacle avoidance for the 4-agent MAS are presented in Figure 3, Figure 4 and Figure 5. A video of this experiment can be seen in the link https://drive.google.com/file/d/1SEDzs_H3UoK7Y2TqMTGHMZ5M3Q16p86q/view (accessed on 24 June 2021). Figure 3 shows snapshots of the motion of the robots in an environment with a fixed obstacle in the form of a column. The images on the left correspond to the initial position of the DDRs in an arbitrary configuration; the images at the center present an intermediate position where one of the DDRs was avoiding the obstacle, and the images on the right show that the desired formation was effectively reached despite that the four agents had to avoid the obstacle in some moment during their motion. This can be appreciated in Figure 4a, where the trajectory followed by each robot and the obstacle position with its security distance are depicted. Since the obstacle was at the center of the environment, the trajectories of the four agents passed close to the obstacle and the robots had to avoid it in different moments. Figure 4b shows the trajectories of the virtual agents (variables $\alpha_{v_{i}}$) during the experiment. The initial virtual positions are marked with × and the final positions with a circle, which appears as a single one around coordinates (0.3,0.5). This demonstrates that consensus of the virtual agents was finally achieved although not all the time these agents moved in consensus due to the transients introduced by the obstacle avoidance task. In Figure 5, it is shown that every robot activated the multitask control (42) at least one time during the experiment and the time interval that such control was active was different for each robot.

**Figure 6:**
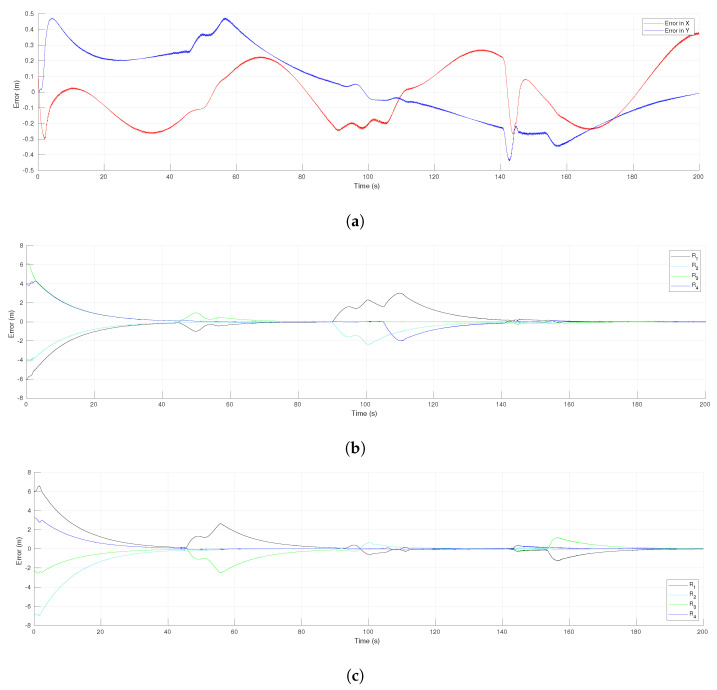
The 4-agent system. Error functions of the formation tracking. (**a**) Tracking errors of the leader. (**b**) Consensus errors in the *x*-axis. (**c**) Consensus errors in the *y*-axis.

Figure 6a shows the evolution in time of the tracking error for the leader $R_{1}$. The leader avoided the obstacle slightly after time 140 s, which was evident in the rapid but continuous change of the tracking error. In other times of the experiment, as around 50 s and 90 s, the effect can be seen of the evasions performed by the followers that affected the tracking error of the reference by the leader; after a transient the tracking error decreased toward zero. The consensus errors of the virtual MAS for coordinates *x* and *y* are shown in Figure 6b,c, respectively. Consensus was achieved in both coordinates in around 45 s for the first time, but after that, the agent $R_{3}$ performed an evasion motion and the consensus error increased for a while, to asymptotically decrease to zero around 90 s. Thus, this effect appeared always that one of the agents avoided an obstacle, due to the use of local information of the network.

**Figure 7:**
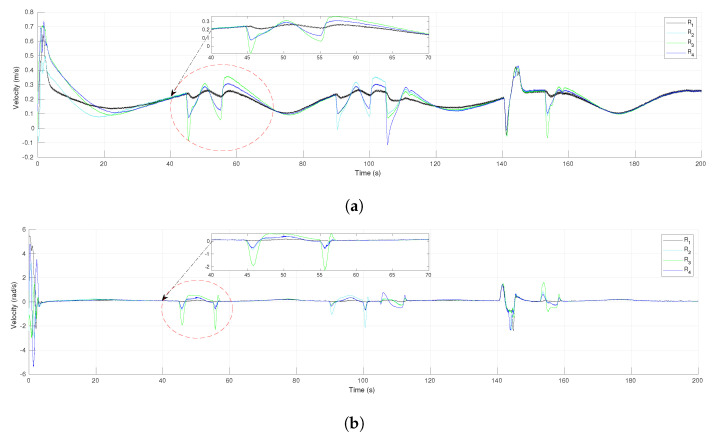
The 4-agent system. Control inputs (velocities) of the DDRs. (**a**) Linear velocities. (**b**) Angular velocities.

The profiles of the control inputs (robot velocities) of each agent are shown in Figure 7a,b, for the linear and angular velocities, respectively. During the first seconds, some important changes were appreciated in the angular velocities due to the DDRs having to align their orientation to the one imposed by the reference trajectory of the leader. It can be seen that all the velocities were the same when the virtual agents are in consensus (the robots move in formation), for instance around 40 s and 85 s. As expected, the evasion actions yielded changes on the velocities evolution, mainly among the follower agents, for instance, the robot $R_{3}$ avoided the fixed obstacle around the time 45 s; it yielded an important change in the velocities of $R_{2}$ and $R_{4}$ and less for the leader $R_{1}$ since the tracking of the reference had a higher weight for the leader’s dynamics (γ>k). Notice that the profiles of velocities were continuous all the time thanks to the use of the smooth transition function h(t).

### 4.2. A 10-Agent System

**Figure 8:**
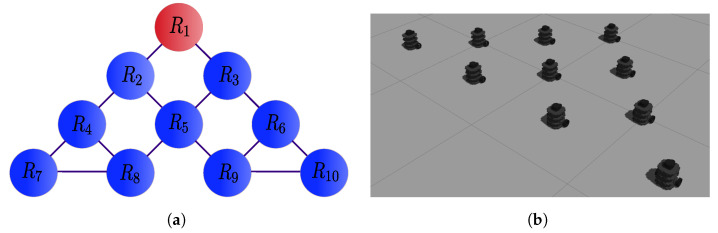
The 10-agent system. (**a**) Connected graph used in the simulations. (**b**) Specified formation in the dynamic simulator Gazebo.

As a second experiment, we considered a 10-agent system in a network with undirected communication topology described by the graph of Figure 8a, with the leader $R_{1}$ as the root. Figure 8b presents the desired formation. We set $a_{ij}=1$ for connected agents and initial conditions $q_{x}\left(0\right)={\left[5,4,6,3.5,5.5,7,3,4.5,6.5,7.5\right]}^{T}$, $q_{y}\left(0\right)=\left(0\right){𝟙}_{N}$ and $q_{\theta }\left(0\right)=\left(\pi /2\right){𝟙}_{N}$ for agents 1 to 10, respectively. The reference trajectory was a circle given by $m\left(t\right)={\left[5\cos(2\pi t/\tau \right),5\sin(2\pi t/\tau )]}^{T}$, with τ=180 s. There were five fixed obstacles in the environment, represented as columns with different diameters in the positions $\alpha_{o_{1}}={\left[-3.5,-2.5\right]}^{T}$, $\alpha_{o_{2}}={\left[-6,-2.5\right]}^{T}$, both with associated security distance $\sigma_{1}=0.4$, $\alpha_{o_{3}}={\left[2,2.5\right]}^{T}$, $\alpha_{o_{4}}={\left[0,-5.8\right]}^{T}$, both with security distance $\sigma_{2}=0.5$ and $\alpha_{o_{5}}={\left[-1,5.3\right]}^{T}$ with $\sigma_{3}=0.6$. The displacement vectors to define the desired formation were $z_{1}={\left[0,1\right]}^{T}$, $z_{2}={\left[-0.5,0.5\right]}^{T}$, $z_{3}={\left[0.5,0.5\right]}^{T}$, $z_{4}={\left[-1,0\right]}^{T}$, $z_{5}={\left[0,0\right]}^{T}$, $z_{6}={\left[1,0\right]}^{T}$, $z_{7}={\left[-1.5,-0.5\right]}^{T}$, $z_{8}={\left[-0.5,-0.5\right]}^{T}$, $z_{9}={\left[0.5,-0.5\right]}^{T}$ and $z_{10}={\left[1.5,-0.5\right]}^{T}$, for $R_{1}$ to $R_{10}$ respectively, with respect to the formation center. The control gains were set to γ=1, λ=0.85 and k=0.35 for tracking, evasion and consensus, respectively.

**Figure 9:**
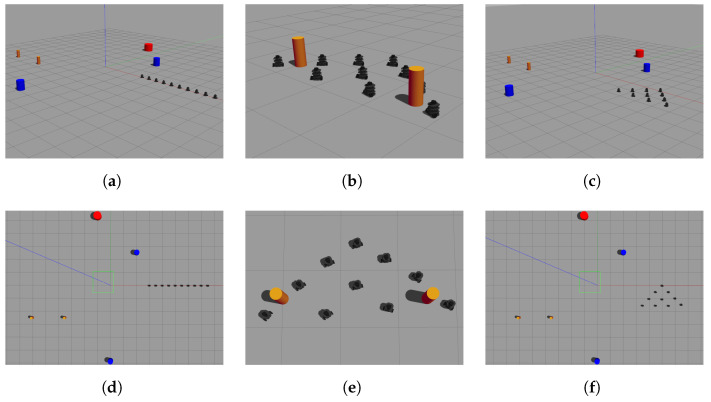
The 10-agent system. Snapshots of the DDRs motion in the simulation. (**a**) Initial position. (**b**) Intermediate position. (**c**) Final position. (**d**) Upper view of initial position. (**e**) Upper view of intermediate position. (**f**) Upper view of final position.

**Figure 10:**
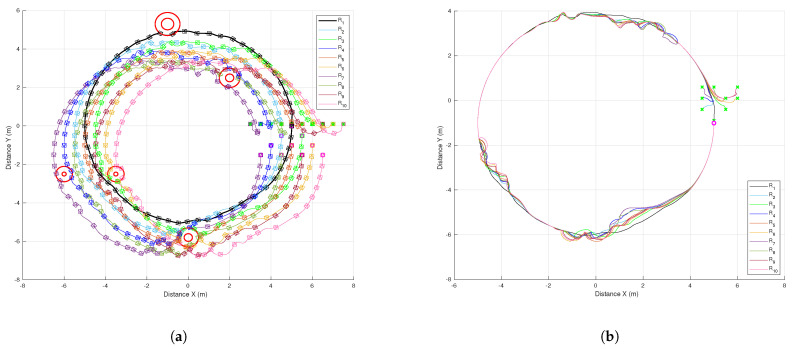
The 10-agent system. Trajectories of the DDRs. (**a**) Trajectories of real agents. (**b**) Trajectories of virtual agents.

**Figure 11:**
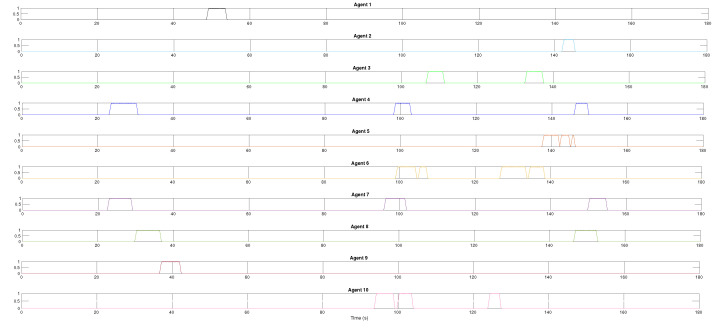
The 10-agent system. Task transition function activation.

**Figure 12:**
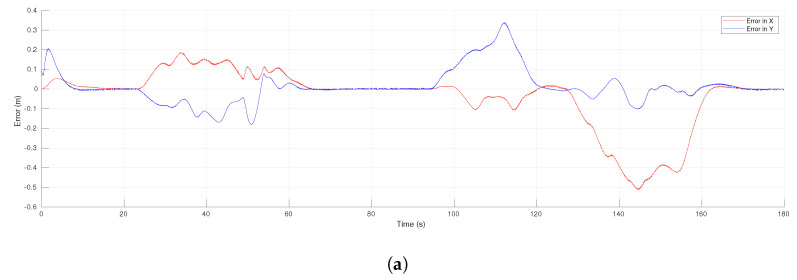

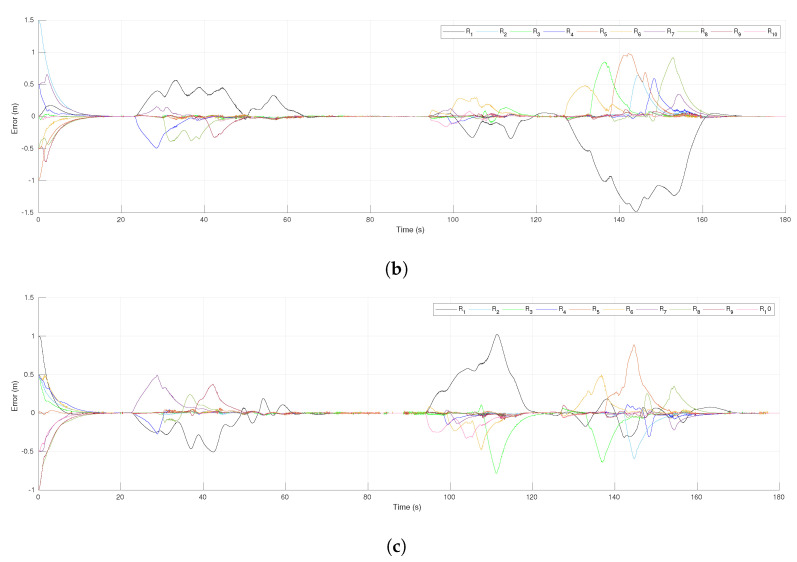
The 10-agent system. Error functions of the formation tracking. (**a**) Tracking errors of the leader. (**b**) Consensus errors in the *x*-axis. (**c**) Consensus errors in the *y*-axis.

**Figure 13:**
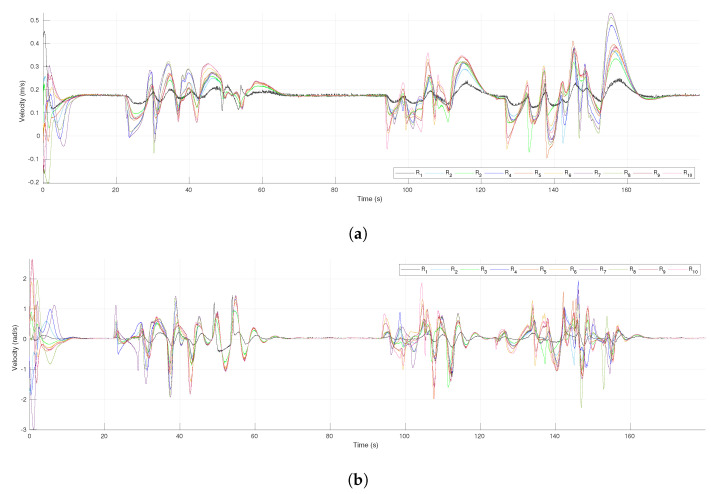
The 10-agent system. Control inputs (velocities) of the DDRs. (**a**) Linear velocities. (**b**) Angular velocities.

The results to implement the proposed distributed control law for the 10-agent MAS are presented in Figure 9, Figure 10, Figure 11, Figure 12 and Figure 13. One can see a video of this experiment in the link https://drive.google.com/file/d/1KoZ4JlW9-lwD5kWcmxN5LNCiIsVQxOZ2/view (accessed on 24 June 2021). Some snapshots of this experiment are shown in Figure 9; the group of robots initiated forming a line and they navigated in the specified triangular formation while obstacles were avoided when needed. The trajectories followed by the robots and the obstacle position with its security distance are depicted in Figure 10a, where one can observe several evasion actions of different agents to avoid collisions with the fixed obstacles and between robots. The trajectories of the virtual agents (variables $\alpha_{v_{i}}$) for this experiment are presented in Figure 10b. It can be seen that the circular trajectory imposed by the leader was well tracked by the 10 agents except when evasion actions were performed, however, after a transient the group of robots returned to consensus. The final consensus value reached when the leader trajectory ended in 180 s was the point (5,−1) marked with a circle.

In the case of the 10-agent system, due to the number of robots and the number of fixed obstacles, many more evasion actions were performed in comparison to the 4-agent experiment. Figure 11 shows the transition function along the simulation time for each robot. All the agents activated the multitask control (42) at least one time during the experiment to avoid collisions with the fixed obstacles whether only one agent had the transition function activated or to avoid other agents whether more than one agent had in high value the transition function. For instance, the leader avoided a fixed obstacle around time 50 s and agents $R_{4}$ and $R_{7}$ avoided each other around time 22 s.

Figure 12a presents the tracking error for the leader $R_{1}$ along the experiment. There was an initial transient since the leader did not initiate over the reference trajectory. In the time intervals from 22 s to 63 s and from 98 s to 165 s, it is clear the effect of the evasion actions performed by the followers that affected the tracking of the reference by the leader, however the tracking error converged to zero after those transients. In the same time intervals could be observed transients due to the evasion actions over the consensus errors of the virtual MAS for coordinates *x* and *y*, presented in Figure 12b,c, respectively. Nevertheless, the consensus errors converged asymptotically to zero after the transients due to evasions and they were null at the final position where the leader stopped at time 180 s. Figure 13 shows the linear and angular velocities of all the robots. As expected, in the same time intervals where evasion actions were performed, the velocities had important changes but they returned to be all the same value during the intervals where the whole group moved in the desired formation, as around 80 s and from 170 s to the end. Furthermore, the velocities were continuous all the time since the function h(t) defined for each robot generated a smooth transition between the control laws of Theorem 1 and the one of Theorem 2.

### 4.3. Environments with Unknown Polygonal Obstacles

**Figure 14:**
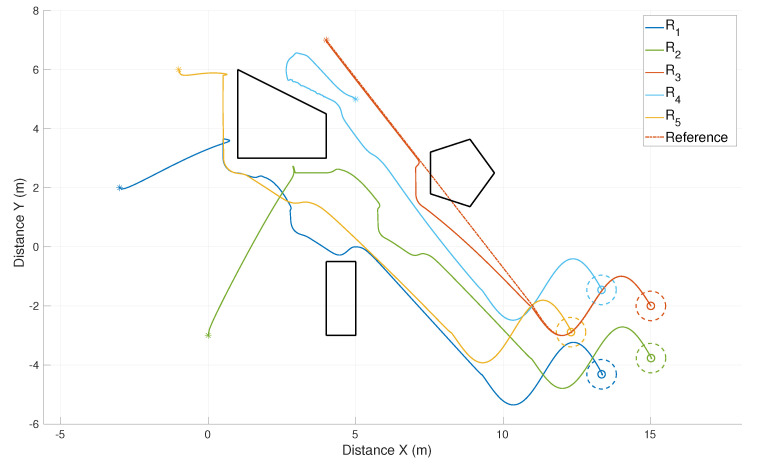
Environment with polygonal obstacles. Trajectories of five robots dealing with irregular unknown obstacles.

**Figure 15:**
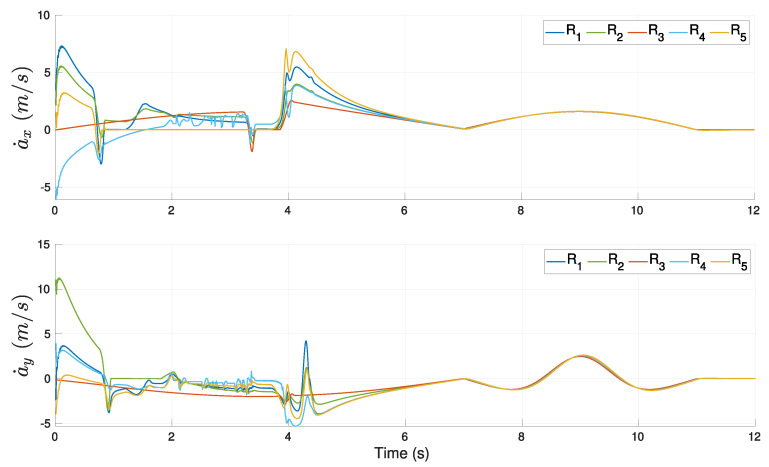
Environment with polygonal obstacles. Velocities of the control points of each robot.

This section presents results of simulations in a different kind of environment in comparison with the previous sections where circular obstacles were considered. In this case, we had a cluttered environment with large irregular obstacles with polygonal shapes. This result was implemented in MATLAB for a rapid prototyping of onboard sensors, since we simulated a scanning range finder for each robot, which allows the robots the detection of the nearest obstacle. When an obstacle was detected within the security distance σ=0.25 the obstacle avoidance task was activated. The simulation setup corresponded to five robots and three obstacles of different shapes. The connectivity graph was circular and the desired formation specified by displacement vectors was a pentagon. The robot $R_{3}$ was the leader, which had to track a linear trajectory starting and finishing in the coordinates (4,7) and (11,−2), respectively. This reference trajectory of the leader continued by following a sinusoidal cycle to finally reach the coordinate (15,−2). Figure 14 shows the trajectories of the control points of each robot starting from arbitrary positions marked with asterisks. It can be seen that the robots avoided the obstacles in the environment and achieved the desired formation as a pentagon. The reference of the leader is depicted as a dash-dotted line trespassing the pentagonal obstacle. The leader effectively avoided this obstacle and returned to track the reference. Figure 15 shows the continuous velocities of the control points ($\alpha_{i}$) of each robot in the *x* and *y* coordinates. The reference trajectory of the leader was designed to start and finish motionless for each part of it. Thus, the linear part of the reference finished in 7 s while the sinusoidal part was performed from 7 to 11 s. After that, the velocities became null and the formation stopped since there was no consensus error among the virtual agents.

### 4.4. Experiments with Real Robots

In this section, the proposed approach was evaluated in experiments with real robots using a group of three DDRs, integrated by a Pioneer 3DX (used as the leader agent) and two TurtleBots 2 (used as the followers). The gains were set to γ=0.8, λ=0.8 and k=0.12 for tracking, evasion and consensus, respectively. The desired formation was triangular, specified by the displacement vectors $z_{1}={\left[0,0\right]}^{T}$, $z_{2}=0.7{\left[\cos\left(\pi /4\right),\cos\left(\pi /4\right)\right]}^{T}$ and $z_{3}=0.7{\left[\cos\left(3\pi /4\right),\sin\left(3\pi /4\right)\right]}^{T}$, with respect to the leader’s $R_{1}$ position. The considered graph was undirected and connected, such that each robot has communication with the other two. Each robot was connected via WiFi to a computer where the velocity commands were computed in a distributed way using ROS. Each agent published its computed velocities and also read the computed controls published by its neighbors at a sampling rate of 0.025 ms. The position of each robot and the obstacles position in the environment were obtained from an Optitrack Motion Capture System.

#### 4.4.1. Linear Trajectory

As a first experiment, the leader performed the tracking of a linear trajectory, which was given by $m\left(t\right)={\left[x,\lambda x+b\right]}^{T}$, where $x=\left(p_{x_{0}}-p_{x_{\tau }}\right)\left(1+\cos\left(\pi t/\tau \right)\right)/2+p_{x_{\tau }}$ with τ=30 s, $\lambda =\left(p_{y_{\tau }}-p_{y_{0}}\right)/\left(p_{x_{\tau }}-p_{x_{0}}\right)$, $b=-\lambda p_{x_{0}}+p_{y_{i}}$ and $\left(p_{x_{0}},p_{y_{0}}\right)$, $\left(p_{x_{\tau }},p_{y_{\tau }}\right)$ were the initial and final coordinates of the trajectory, respectively. Besides, a fixed obstacle was at the position $\alpha_{o}={\left[0.91,0.12\right]}^{T}$ with security distance σ=0.4.

**Figure 16:**
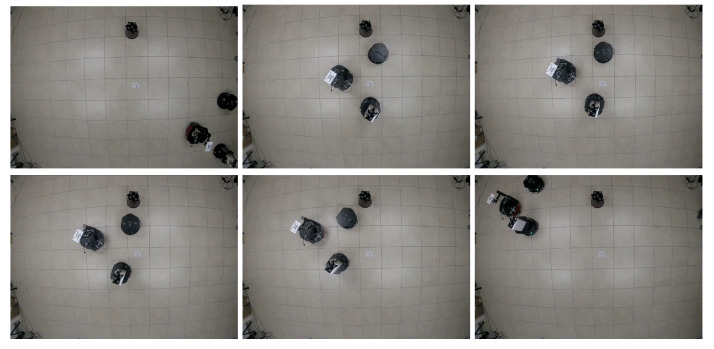
Three-agent system linear trajectory. Snapshots of the robots motion in the experiment. The initial configuration is at the top left and the final formation is at the bottom right. Intermediate positions when an obstacle is avoided are shown.

**Figure 17:**
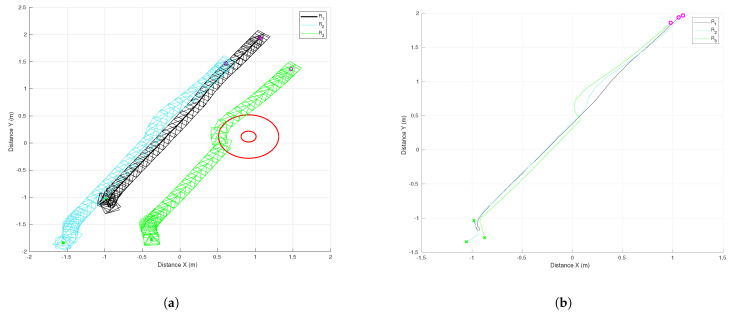
Three-agent system linear trajectory. (**a**) Trajectories of real agents. (**b**) Trajectories of virtual agents.

**Figure 18:**
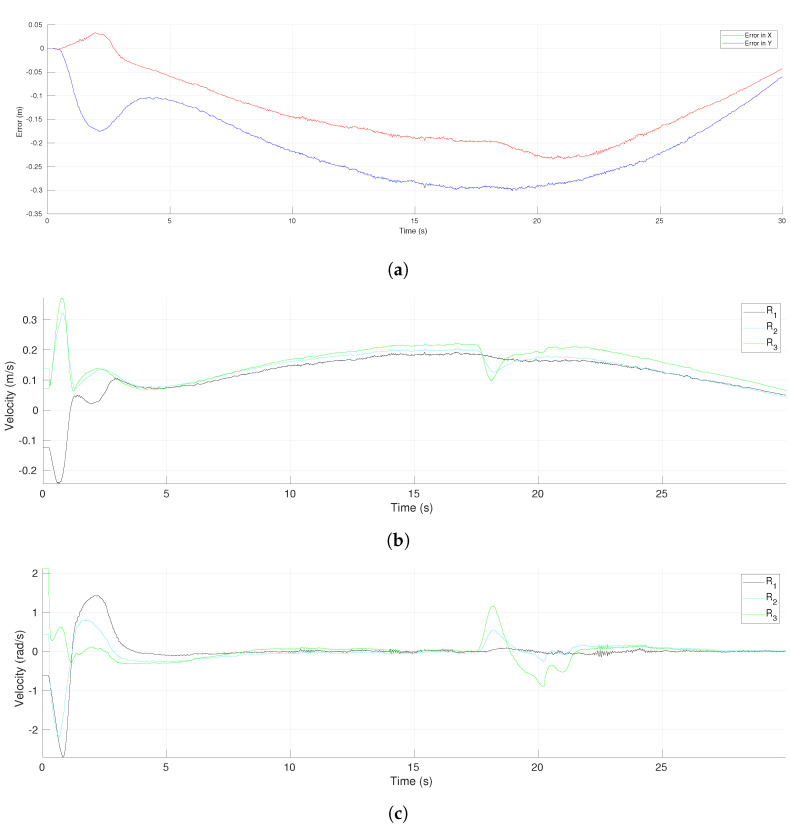
Three-agent system linear trajectory. (**a**) Tracking errors of the leader. (**b**) Linear velocities. (**c**) Angular velocities.

The results of this experiment are presented in Figure 16, Figure 17 and Figure 18 and the corresponding video can be seen in the link https://drive.google.com/file/d/1RVNe52Gh6gmc2AtGyeuERfKY54dixHz-/view (accessed on 24 June 2021). Figure 16 shows some snapshots of the video with the initial configuration at the top left and the final formation at the bottom right. Some intermediate positions are presented where one of the DDRs was avoiding the obstacle and also caused the others to move. This was clear in the trajectories of the robots as given by the motion capture system that can be observed in Figure 17a. The position of the obstacle is also shown with its security distance depicted as a red circle. Figure 17b shows the trajectories of the virtual agents computed from the position of the robots and the displacement vectors $z_{i}$. One can observe that the robots moved in formation until $R_{3}$ had to avoid the obstacle, which generated a reaction over the motion of $R_{2}$ and a small effect on the leader $R_{1}$. At time 30 s, the virtual agents reached consensus close to the coordinate (1.1,2), which was the final point of the reference of the leader.

The evolution of the tracking errors for the leader $R_{1}$ with respect to time is shown in Figure 18a. One can observe an initial transient and a maximum error slightly after the middle of the time, which was expected given the form of the reference trajectory with maximum velocity in the middle of the trajectory. After that middle point, the errors in both coordinates were reduced to reach low final values. The effect of the obstacle avoidance performed by $R_{3}$ could be appreciated around time 18 s with a smooth change in the *x*-coordinate of the tracking error. Figure 18b,c show the profiles of the computed robot velocities (linear and angular, respectively) for each agent. An initial transient was appreciated during the first 5 s and after that, the velocities evolved very close each other until the robot $R_{3}$ performed an evasion around time 18 s. This action yielded a larger change in the velocities of $R_{3}$ than for $R_{2}$ and had a low effect in the leader’s velocities. The use of the transition function h(t) allowed the proposed scheme to compute continuous velocities along the experiment.

#### 4.4.2. Circular Trajectory

As a second experiment, we considered a circular trajectory, which was parameterized as $m\left(t\right)=1.1{\left[\cos\left(2\pi t/\tau \right),\sin\left(2\pi t/\tau \right)\right]}^{T}$, with τ=70 s, such that the radius of the circle was 1.1 m and the trajectory started at the coordinate (1.1,0) and returns to the same point in τ s. Besides, a fixed obstacle was at the position $\alpha_{o}={\left[0.6,-2.0\right]}^{T}$, with security distance σ=0.55.

**Figure 19:**
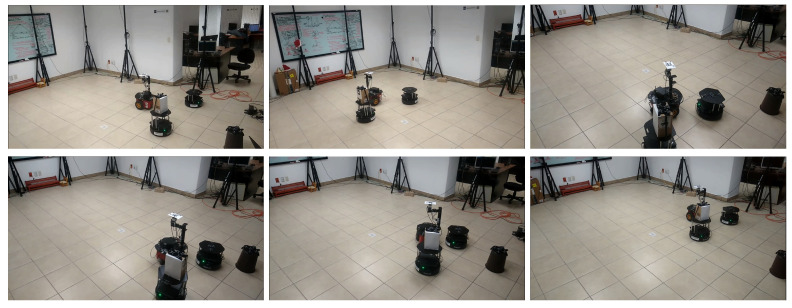
Three-agent system circular trajectory. Snapshots of the robots motion in the experiment. The initial configuration is at the top left and the final formation is at the bottom right. Intermediate positions when an obstacle is avoided are shown.

**Figure 20:**
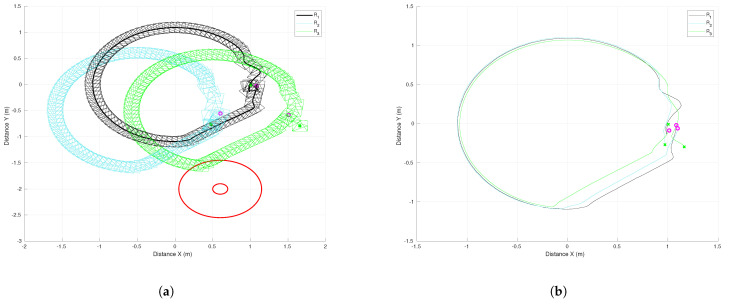
Three-agent system circular trajectory. (**a**) Trajectories of real agents. (**b**) Trajectories of virtual agents.

**Figure 21:**
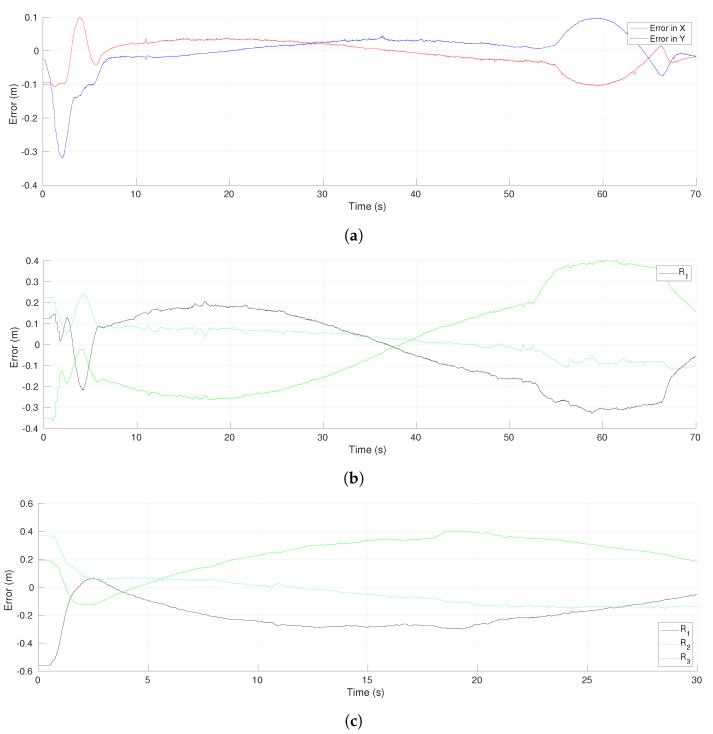
Three-agent system circular trajectory. Error functions of the formation tracking. (**a**) Tracking errors of the leader. (**b**) Consensus errors in the *x*-axis. (**c**) Consensus errors in the *y*-axis.

Figure 19, Figure 20 and Figure 21 present the results of this experiment and the corresponding video can be seen in the link https://drive.google.com/file/d/1bJZBO7dByTipDryC0XqHJurHE4f-d_i1/view?usp=sharing (accessed on 24 June 2021). Some snapshots of the video are presented in Figure 19. The initial configuration of the MAS is the image at the top left and the final formation is at the bottom right. Some intermediate positions are presented; in particular we show the evasion of the fixed obstacle, which causes a deviation of the three robots to later recover the trajectory. Figure 20a depicts the trajectories of the robots and shows how the robot $R_{2}$ performed the evasion when it overstepped the security region. The effect of the obstacle avoidance is also observed in Figure 20b, where the trajectories of the virtual agents are shown. They moved in consensus until the robot $R_{2}$ deviated to evade the obstacle. In this case, the deviation of the evading robot was more significant than in the previous experiment and then the three robots deviated in a similar way. At the end of the trajectory, at 70 s, the virtual agents returned to consensus close to the the final point (1.1,0) of the leader’s reference.

Figure 21a shows the evolution of the tracking errors for the leader $R_{1}$ with respect to time. After the initial transient, the errors remained at low values and they had a second transient due to the effect of the obstacle avoidance around time 53 s. Both coordinates were affected in this case, but at the end the errors converged asymptotically to zero as proven in Theorem 2. The consensus errors of the virtual MAS for coordinates *x* and *y* are shown in Figure 21b,c, respectively. The consensus errors were also maintained at low values and they increased around time 53 s due to the evasion task initiated by robot $R_{3}$. The avoidance task finished around time 65 s and then the consensus errors converged to reach a low value that allowed the MAS to return to the desired formation. Since the robot velocities depended on the errors shown in these figures, the proposed scheme provided continuous velocities along the experiment thanks to the smooth switching of control laws.

## 5. Conclusions and Discussion

In this paper, we have proposed a distributed control scheme to solve the problem of position formation of a group of differential-drive robots while tracking a predefined trajectory guided by a leader, all moving in an environment with obstacles. The obstacles can be the agents themselves or fixed objects in the environment. The proposed scheme is valid for connected communication topologies. A hierarchical task-based scheme has been introduced for the formation tracking problem with obstacle avoidance. Thus, whether a robot is close to an obstacle at a given distance, the task with higher priority is devoted to avoid the obstacle and the task with lower priority is the formation tracking, and whether no robots are near to the obstacles only the formation tracking control is executed.

We have formulated an adequate task function for the formation tracking and has been combined to solve both tasks simultaneously when needed, guaranteeing continuity of the velocities computed by the control law, independently of the activation/deactivation of the obstacle avoidance task. The proposed control scheme only needs relative information between neighboring agents and local measurements to avoid obstacles, in such a way that neither a global coordinate system nor a map of the environment are required. The convergence of the proposed control scheme to the formation tracking in spite of the execution of evasion actions is proven theoretically and its effectiveness is validated through realistic simulations and experiments using real robots.

As future work, we want to extend the proposed hierarchical scheme to include more tasks, for instance connectivity maintenance (focused on preserve the initial connected graph or guarantee the maintenance of a connected graph regardless of changes in the communication topology), consider more than one leader and different formations in sub-groups, as well as the implementation of the approach for long-distance navigation experiments in more general environments with complex obstacles. To do so, we will focus on developing experimental work relying only on local measurements using onboard sensing, such as laser range finders or depth cameras mounted on the robots, instead of a global positioning system.

## Data Availability

Not applicable.
